# Activation of Hippocampal IR/IRS-1 Signaling Contributes to the Treatment with Zuogui Jiangtang Jieyu Decoction on the Diabetes-Related Depression

**DOI:** 10.1155/2021/6688723

**Published:** 2021-06-03

**Authors:** Hui Yang, Jia Ling, Pan Meng, Jian Liu, Xiaoyuan Lin, Wei Li, Yuhong Wang

**Affiliations:** ^1^The First Hospital of Hunan University of Chinese Medicine, Changsha, Hunan, China; ^2^Key Laboratory of Chinese Material Medical Power and Innovation Drugs Established By Provincial and Ministry, Hunan University of Chinese Medicine, Changsha, Hunan, China

## Abstract

**Background:**

Zuogui Jiangtang Jieyu decoction (ZJJ) is mainly used for the treatment of diabetes-related depression in current clinical applications and research. This study aims to investigate whether the brain IR/IRS-1 signaling pathway is involved in the therapeutic effect of ZJJ on depression-like behavior in diabetic rats.

**Methods:**

Sprague–Dawley rats were fed with high-fat diet and subjected to streptozotocin injection to establish the diabetes animal model. After treatment with different doses of ZJJ (20.530 g/kg or 10.265 g/kg) for 4 weeks, the blood glucose level and peripheral insulin resistance were measured. The forced-swimming test (FST) and Morris water maze test (MWMT) were applied for the mood and cognitive function assessment. Then, the Western blot method was used to analyze the protein levels of insulin receptor (IR), insulin receptor substrate-1 (IRS-1), phosphatidylonositol-3-kinase (PI3K), and protein kinase B (PKB, also as known as AKT) in the hippocampus of diabetic rats. Meanwhile, the immunofluorescence method was performed to analyze the above proteins' expression in the neuron and astrocyte. At last, the levels of glycogen, lactate, and ATP were tested by the ELISA method. Additionally, the insulin-sensitive glucose transporter 4 (GLUT4) and the lactate transporter monocarboxylate transporter 4 (MCT4) were analyzed by the Western blot method.

**Results:**

ZJJ administration significantly decreased the level of blood glucose and improved the peripheral insulin resistance in diabetic rats. Besides, ZJJ attenuated the depression-like behavior and the cognitive dysfunction in rats with diabetes. Furthermore, we found the upregulation of protein expression of phospho-IR, phospho-IRS-1, phospho-PI3K, and phospho-AKT in the hippocampus of diabetic rats after being treated with ZJJ. Moreover, the above proteins are increased not only in the neuron but also in the astrocyte after ZJJ administration. In addition, ZJJ increased the content of ATP, glycogen, and lactate, as well as the expression of GLUT4 and MCT4 in the hippocampus of diabetic rats.

**Conclusions:**

These findings suggest that ZJJ improves the depression-like behavior of diabetic rats by activating the IR/IRS-1 signaling pathway in both hippocampal neuron and astrocyte. And the brain IR/IRS-1 signaling pathway plays an important role in astrocyte-neuron metabolic coupling, providing a potential mechanism by which the IR/IRS-1 signaling pathway may contribute to the treatment of ZJJ on diabetes-related depression.

## 1. Introduction

Depressive symptoms, including sadness, cognitive dysfunction, disrupted sleep, appetite changes, or suicidality, may occur in people with diabetes [1]. Subsequently, depression can exacerbate and accelerate adverse clinical profiles of diabetes [2]. However, the underlying molecular mechanism between diabetes and depression remains elusive. A clinical report indicates that individuals with diabetes and insulin resistance have a higher risk of suffering from depression [3]. Conversely, patients with the major depressive disorder were 1.5 times more likely to develop insulin resistance than people without depression [4]. In fact, insulin resistance, as a hallmark of diabetes, exists not only in the periphery but also in the brain. And the defective brain insulin signaling may induce depression-like behavior by regulating the processes of neurogenesis, energy metabolism, neuroinflammation, etc. Thus, brain insulin resistance may play a critical role in the development of diabetes-related depression [5].

Insulin and its specific receptor (insulin receptor, IR) are highly expressed in the hippocampus, which is the core area of emotional regulation and cognitive abilities. And the binding between insulin and IR leads to recruitment and phosphorylation of the insulin receptor substrates 1 (IRS-1), which then activates the PI3K/AKT pathway. Furthermore, the PI3K/AKT pathway applies to downstream nodes, such as glycogen synthase kinase 3*β* (GSK-3*β*) [6]. The above molecules regulate the survival and conduction functions of hippocampal neurons. A previous study indicated that an abnormal IR/IRS-1 signaling in the hippocampus was closely related to the cognitive disorder caused by diabetes, while the PI3K/AKT signal in the hippocampus could also regulate emotional impairment [7]. And an array of metabolic abnormalities and mental disorders were observed when the insulin receptor (IR) in the brain was knockdown [8]. Therefore, the neuronal IR/IRS-1 signaling pathway and its downstream PI3K/AKT signaling may play a key role in diabetes-related depression.

Although most studies suggested that the cell responding to insulin in the hippocampus is mainly neurons, a recent study showed that insulin can act on astrocytes (a gliocyte in the hippocampus) and produce physiological effects [9]. The astrocyte is a “hopeful” target for intervention of emotional abnormalities [10]. The clinical study reveals that the astrocyte is relevant to volume loss in the hippocampus in subjects with major depression [10]. The mice showed the anxiety and depression-like behavior when IR was knockdown in the astrocyte [9]. In fact, the damage of astrocytes is not only existed in depression but also found in mood disorder and cognitive impairment in diabetes. The expression of glial fibrillary acidic protein (GFAP) was increased in the hippocampus of streptozotocin (STZ)-induced diabetic mice, which accompanied with deficits on cognitive function [11]. Consequently, IR/IRS-1 signaling in the astrocyte may involve in the comorbid depression in diabetes.

Zuogui Jiangtang Jieyu decoction (ZJJ) is a Chinese herbal formulation on the strength of Zuogui Wan, which was recorded in the “Jing Yue Quan Shu” in the Ming dynasty. Our previous studies have reported the hypoglycemic and antidepressant effect of ZJJ [12, 13]. And it also shows the ability to regulate the level of glucocorticoid in diabetic rats with depression [13]. Given that diabetes-induced brain insulin resistance is associated with the elevated glucocorticoid level caused by alteration of HPA axis [14], the effects of ZJJ on diabetes-related depression may be related with the hippocampal IR/IRS-1 signaling. Thus, in this study, we investigated the effect of ZJJ on the IR/IRS-1 signaling pathway in the hippocampal neuron and astrocyte.

## 2. Materials and Methods

### 2.1. Drugs and Reagents

ZJJ was provided by the First Hospital of Hunan University of Chinese Medicine. It consists of eleven herbs—namely, *Astragalus membranaceus* (Fisch.) Bge. (astragali radix, 18 g), *Hypericum perforatum* L. (hyperici perforati herba, 3 g), *Curcuma longa* L. (curcumae longae rhizoma, 9 g), *Rehmannia glutinosa* Libosch. (rehmanniae radix praeparata, 15 g), *Cornus officinalis* Sieb. et Zucc. (corni fructus, 12 g), *Lycium barbarum* L. (lycii fructus, 12 g), *Cuscuta australis* R. Br. (cuscutae semen, 9 g), *Eucommia ulmoides* Oliv. (eucommiae cortex, 9 g), *Salvia miltiorrhiza* Bge. (salviae miltiorrhizae radix et rhizoma, 12 g), *Paeonia suffruticosa* Andr. (moutan cortex, 6 g), and *Achyranthes bidentata* Bl. (achyranthis bidentatae radix, 9 g).

### 2.2. Animal Materials

Six-week-old male Sprague–Dawley (SD) rats, weighing 180 to 200 g, were purchased from the Hunan Slack Scene of Laboratory Animal Company (Hunan, China). All rats were maintained in the SPF Laboratory Animal Center in the First Affiliated Hospital of the Hunan University of Chinese Medicine. The temperature in the room is maintained at 23 ± 1°C with a 12 h light/dark cycle. All laboratory procedures were performed according to the guidelines established by the Animal Ethics Welfare Committee of the First Affiliated Hospital of the Hunan University of Chinese Medicine.

### 2.3. Experimental Groups and Drug Administration

Rats were fed with high-fat diet (HFD) for four weeks to inducing obesity, and then they were injected with STZ (38 mg/kg, i.p.), which dissolved in sodium citrate buffer (pH 4.9, 4°C) to establish diabetes. Rats were considered diabetic if the fasting blood glucose levels >16 mmol/L. Then, the experimental subjects were divided into 5 groups, consisting of control group (Control), diabetic group (DM), metformin (1.8 mg/kg) and fluoxetine (10.8 mg/kg) group (MET/FLX), high-dose (20.530 g/kg) Zuogui Jiangtang Jieyu decoction group (ZJJ-H), and low-dose (10.265 g/kg) Zuogui Jiangtang Jieyu decoction group (ZJJ-L). Treatment groups were administrated with ZJJ or MET/FLX for 4 weeks starting from the eighth week. After the treatment, the depression-like behavior and cognitive function of rats were tested by the forced-swimming test (FST) and Morris water maze test (MWMT), respectively.

### 2.4. Animal Behavior Test

#### 2.4.1. Forced-Swimming Test (FST)

The forced-swimming test (FST) was performed to assess the depression-like behavior of rats [15]. In this experiment, rats were placed in a circular fiberglass pool, containing 30 cm of water at 25 ± 1°C. The duration of observed immobility time was recorded during the last 3 min of the whole 4 min testing period. And decreased immobility time was used as the index of antidepressant-like efficacy.

#### 2.4.2. Morris Water Maze Test (MWMT)

The Morris water maze test was used to assess the cognitive function of rats in this study [15]. Each rat was placed in a cylinder water tank (200 cm in diameter) filled with water (25 ± 1°C). And the container was divided into four equal quadrants. For the place navigation test, rats were daily introduced to a different quadrant on the first four days. And the time of rats to find the submerged platform was recorded as the escape latency time, which was used as the index of learning function. In the spatial probe test, rats were placed in the water tank where the platform was removed. The time spent in the target quadrant by rats and the number of times that the rats crossed the platform area were recorded to assess the memory function of rats.

### 2.5. ELISA Analysis

Fasting blood glucose (FBG) was detected by using fasting blood samples from rat tail veins. The levels of fasting insulin (FINS) (Nanjing Jiancheng, China), ATP (Nanjing Jiancheng, China), glycogen (Feiya Biotechnology, China), and lactate (Feiya Biotechnology, China) were tested by using enzymatic kits according to the manufacturer's instructions. The assessment of insulin resistance was calculated as followed: (HOMA-IR) = (FBG × FINS)/22.5. HOMA-IR refers to the homeostasis model assessment of insulin resistance.

### 2.6. Western Blot Analysis

Western blot analysis was performed as described in our previous study [13]. Briefly, equal amounts of proteins were separated by SDS-PAGE and transferred to polyvinylidene fluoride (PVDF) membranes (Millipore Corporation, USA). After the membranes were blocked by TBST (TBS plus 0.05% Tween 20) plus 1% nonfat dry milk for 60 min, proteins were incubated with primary antibody overnight at 4°C. The following antibodies were used: anti-IR (1 : 1000; Affinity biosciences, USA), anti-phospho-IR (1 : 2000; Affinity biosciences, USA), anti-IRS-1 (1 : 1000; Cell signaling, USA), anti-phospho-tyr-IRS-1 (1 : 1000; Cell signaling, USA), anti-PI3K (1 : 1000; Affinity biosciences, USA), anti-phospho-PI3K (1 : 2000; Affinity biosciences, USA), anti-AKT (1 : 1000; Cell signaling, USA), anti-phospho-AKT (1 : 2000; Affinity biosciences, USA), anti-MCT4 (1 : 2000; Proteintech, USA), anti-GLUT4 (1 : 1000; Proteintech, USA), and anti-GAPDH (1 : 1000, Cell signaling, USA). PVDF membranes were then washed by TBST and incubated with HRP-linked antibody(1 : 3000; Cell signaling, USA) at room temperature for 60 min. PVDF membranes were then washed by TBST and developed using Enhanced Chemiluminescence Reagents (ECL; New Cell & Molecular Biotech, China). At last, the protein bands were analyzed by the Image Lab system.

### 2.7. Immunofluorescence Analysis

Rats were perfused sequentially with (1) 250 mL of saline solution and (2) 250 mL of 4% paraformaldehyde in 0.1 M phosphate buffer saline (PBS). The slices of brain were cut by a microtome and incubated with primary antibody for the phospho-IR (1 : 200; Affinity Biosciences, USA), phospho-IRS-1 (1 : 200; Bioss Antibodies, China), phospho-PI3K (1 : 100; Affinity Biosciences, USA), phospho-Akt (1 : 100; Affinity Biosciences, USA), or GFAP (1 : 200; Cell Signaling, USA). Then, sections were washed and incubated with anti-IgG secondary antibody coupled to FITC (green) or CY3 (red). Tissue sections were examined on a fluorescence microscope (Nikon Eclipse C1).

### 2.8. Statistical Analysis

All the data were presented as mean ± standard error of the means (S.E.M.). Two-way ANOVA was performed to detect the differences between two ggroups, and Tukey's post hoc analysis was performed when appropriate. *P* values < 0.05 was considered to be statistically significant.

## 3. Results

### 3.1. ZJJ Treatment Improves Peripheral Insulin Resistance in Diabetic Rats

**Figure 1:**
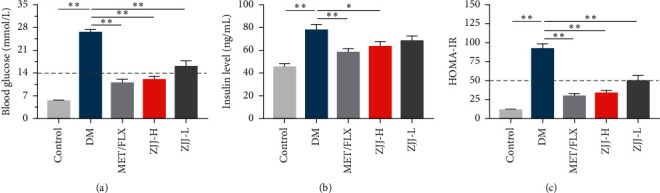
Effect of ZJJ on peripheral insulin resistance in diabetic rats. (a) Both high and low doses of ZJJ reduced the levels of blood glucose. (b) High dose of ZJJ reduced the levels of blood insulin. (c) Both high and low doses of ZJJ reduced the value of HOMA-IR. *n* = 8 in each group. ^*∗∗*^*P* < 0.01 and ^*∗*^*P* < 0.05. Data are presented as the mean ± S.E.M. HOMA-IR: homeostasis model assessment of insulin resistance.

The effect of ZJJ on peripheral glucose metabolism was investigated in diabetic rats. As shown in Figure 1, the levels of blood glucose and insulin significantly increased in diabetic rats when compared with those in the control group (*P* < 0.01). The high dose (20.530 g/kg) of ZJJ effectively decreased the blood glucose and insulin levels (*P* < 0.01), while the low dose (10.265 g/kg) only lowered the blood glucose level in diabetic rats (*P* < 0.05). Furthermore, two doses of ZJJ (20.530 g/kg and 10.265 g/kg) treatment significantly reduced the value of HOMA-IR in diabetic rats (*P* < 0.01).

### 3.2. ZJJ Treatment Attenuates Depression-Like Behavior and Cognitive Dysfunction in Diabetes Rats

**Figure 2:**
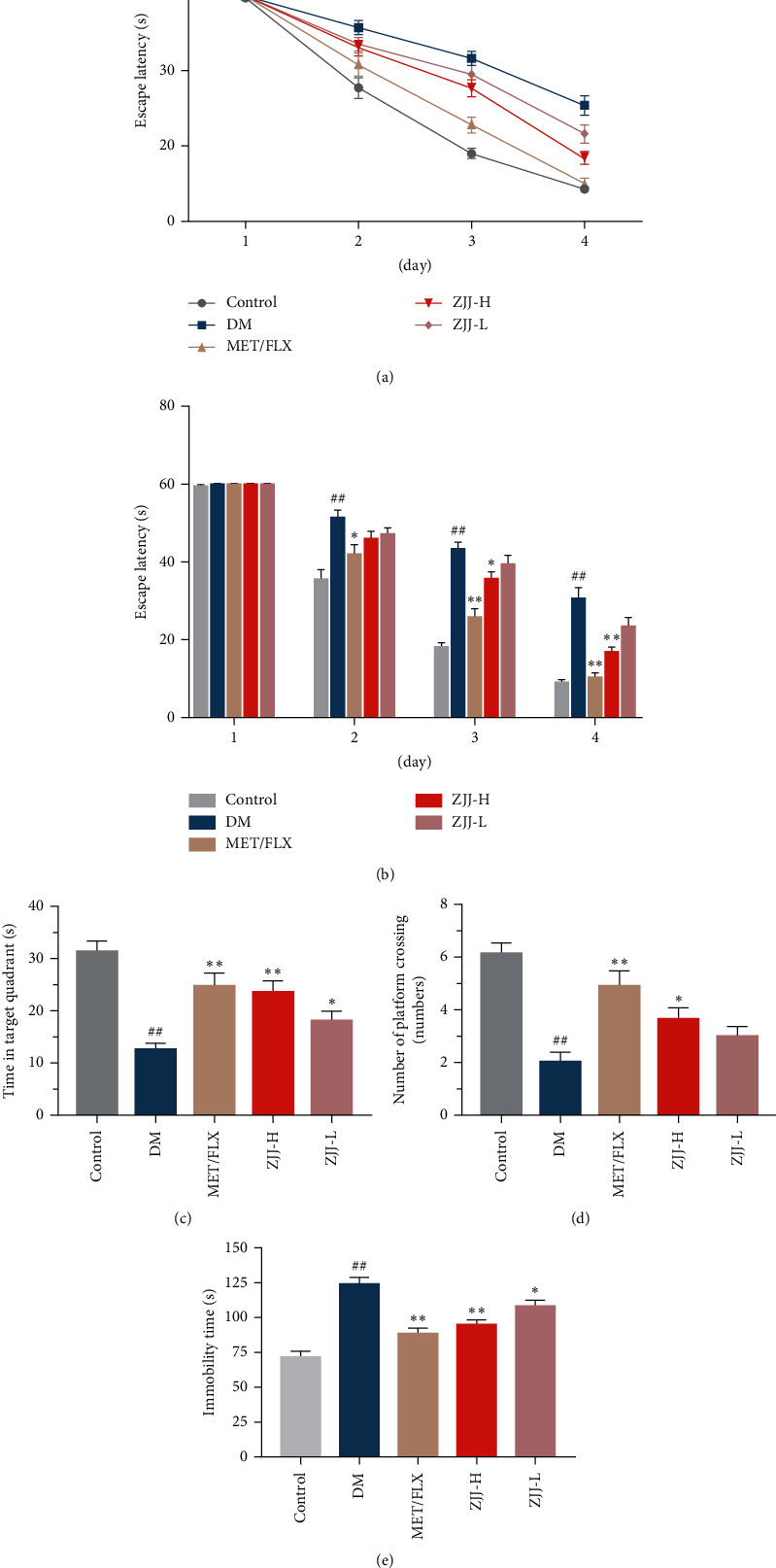
ZJJ ameliorated the depression-like behavior and cognitive function in diabetic rats. (a) The cognitive function of rats was evaluated by the Morris water maze test, and the learning function of rats was analyzed in the part of place navigation test. The escape latency of rats in all groups decreased during four training days. (b) The high dose of ZJJ treatment decreased the escape latency time on days 3 and 4 in diabetic rats. (c) The memory function of rats was measured in the part of spatial probe test. Both doses of ZJJ increased the swimming time of diabetic rats in the target quadrant. (d) ZJJ increased the times of rats crossing the platform. (e) The depression-like behavior of rats was evaluated by the forced-swimming test, and the both doses of ZJJ reduced the immobility time of diabetic rats. *n* = 8 in each group. ^##^*P* < 0.01 vs. the control group. ^*∗∗*^*P* < 0.01 and ^*∗*^*P* < 0.05 vs. the DM group. Data are presented as the mean ± SEM.

The effect of ZJJ on cognitive performance (including learning and memory) and mood was investigated by the Morris water maze test and forced-swimming test, respectively. In the Morris water maze test, as shown in Figure 2(a), the escape latency decreased during four training days in all groups. More concretely, there was no significantly different escape latency time between the control group and DM group on the first training days, while the DM rats show a significantly increased latency time on the last three days compared with the normal rats (Figure 2(b), *P* < 0.01). Treatment of the high dose of ZJJ (20.530 g/kg) obviously reduced the latency time of diabetic rats on days 3 and 4 during training trials (Figure 2(b), day 3: *P* < 0.05, and day 4: *P* < 0.01). Furthermore, ZJJ treatment increased the swimming time in target quadrant (Figure 2(c), 20.530 g/kg: *P* < 0.01, and 10.265 g/kg: *P* < 0.05) and the times that rats crossing the platform (Figure 2(d), 20.530 g/kg: *P* < 0.05). The above results suggest that ZJJ improved the cognitive function of diabetic rats. In addition, the reduced immobility time in force swimming is used as the index of antidepressant-like efficacy. And it was dramatically increased in the DM group when compared with the control group (Figure 2(e), *P* < 0.01). Conversely, both doses of ZJJ are witnessed with an obvious decrease in the immobility time (Figure 2(e), 20.530 g/kg: *P* < 0.01, and 10.265 g/kg: *P* < 0.05).

### 3.3. ZJJ Treatment Activates the Hippocampal IR/IRS-1 Signaling in Diabetic Rats

**Figure 3:**
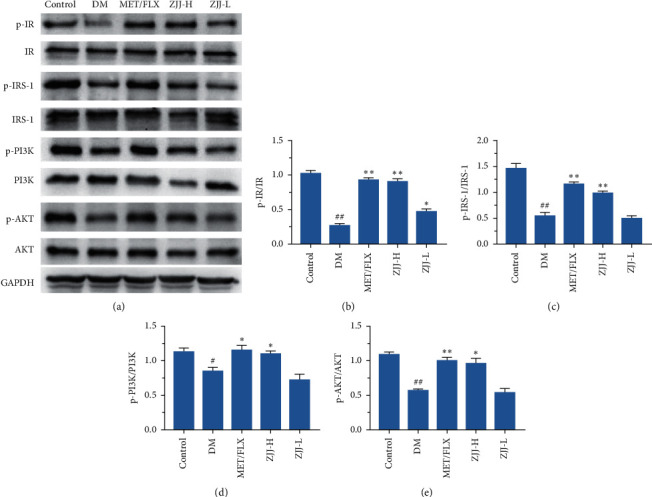
ZJJ activated the hippocampal insulin signaling pathway in diabetic rats. (a) Bands of p-IR, IR, p-IRS-1, IRS-1, p-PI3K, PI3K, p-AKT, AKT, and GAPDH. (b–e) Both doses of ZJJ treatment increased the expression of p-IR (b), while the high dose of ZJJ treatment increased the expression of p-IRS-1 (c), p-PI3K (d), and p-AKT (e) in diabetic rats. *n* = 6 in each group. ^##^*P* < 0.01 and ^##^*P* < 0.05 vs. the control group. ^*∗∗*^*P* < 0.01 and ^*∗*^*P* < 0.05 vs. the DM group. Data are presented as the mean ± SEM.

The IR/IRS-1 signaling pathway was recognized as an association between diabetes and depression [16]. And aberrant IR/IRS-1 signaling might be related to mood and cognitive impairment [17]. In this study, Western blot analyses show that compared with control rats, the phosphorylation of IR, IRS-1, PI3K, and AKT was significantly decreased in the hippocampus of diabetic rats (Figure 3, *P* < 0.05 or *P* < 0.01). And the reduction of these proteins was improved by treatment with the high dose (20.530 g/kg) of ZJJ (*P* < 0.05 or *P* < 0.01).

### 3.4. ZJJ Treatment Improves the Neuronal IR/IRS-1 Signaling in the Hippocampus of Diabetic Rats

**Figure 4:**
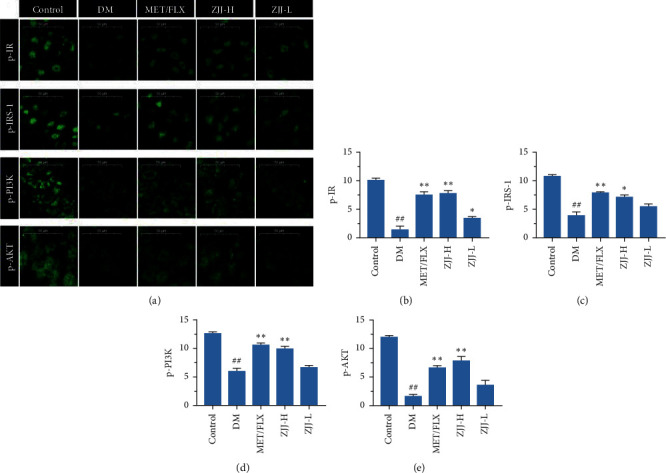
ZJJ activated the neuronal insulin signaling pathway in diabetic rats. (a) Immunofluorescence analysis of p-IR, p-IRS-1, p-PI3K, and p-AKT. (b–e) Both doses of ZJJ treatment increased the immunopositivity of p-IR (b), while the high dose of ZJJ treatment increased the expression of p-IRS-1 (c), p-PI3K (d), and p-AKT (e) in the hippocampus of diabetic rats. *n* = 5 in each group. Bar = 50 *μ*m. ^##^*P* < 0.01 vs. the control group. ^*∗∗*^*P* < 0.01 and ^*∗*^*P* < 0.05 vs. the DM group. Data are presented as the mean ± SEM.

To further investigate the regulation of ZJJ on neuronal IR/IRS-1 signaling, the immunofluorescence analysis was used in this study. As shown in Figure 4, the expressions of p-IR, p-IRS-1, p-PI3K, and p-AKT were significantly decreased in the diabetic rats (*P* < 0.01). And 20.530 g/kg ZJJ treatment increases the above four proteins levels, while 10.265 g/kg ZJJ only increases the expression of p-IR (*P* < 0.01 or *P* < 0.05).

### 3.5. ZJJ Treatment Regulates the Neuronal Energy Metabolism in Diabetic Rats

**Figure 5:**
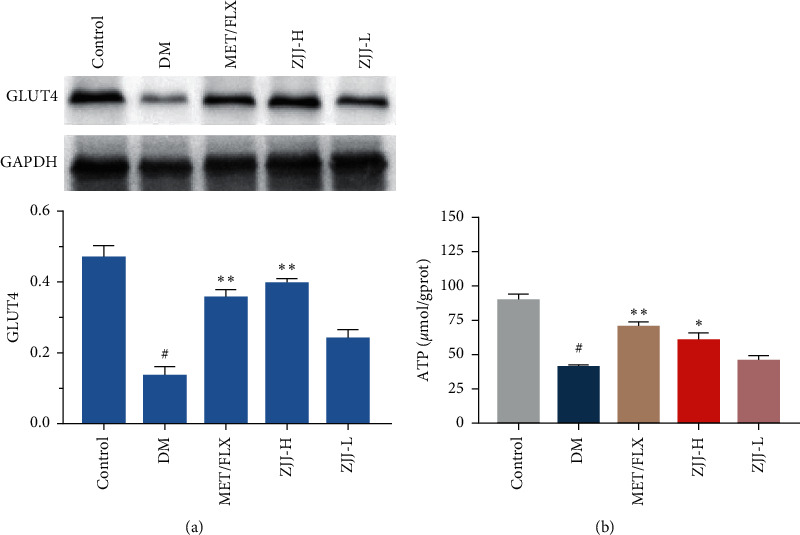
ZJJ improved the neuronal energy metabolism in diabetic rats. (a) Bands of GLUT4 and GAPDH were shown, and the expression of GLUT4 was increased after the high dose of ZJJ treatment in diabetic rats. (b) High dose of ZJJ increased the ATP release (b) in the hippocampus of diabetic rats. *n* = 6 in each group. ^#^*P* < 0.05 vs. the control group. ^*∗∗*^*P* < 0.01 and ^*∗*^*P* < 0.05 vs. the DM group. Data are presented as the mean ± SEM.

Brain IR/IRS-1 signaling in the hippocampus may contribute to neuronal activity by effecting glucose utilization and energy metabolism [18]. In this study, GLUT4, an insulin-sensitive glucose transporter, was obviously reduced in the hippocampus of diabetic rats, and the ATP release was decreased as well (Figure 5, *P* < 0.05). ZJJ treatment (20.530 g/kg) significantly increases the expression of GLUT4 (*P* < 0.05) and the level of ATP (*P* < 0.01), suggesting its potential effect on neuronal energy metabolism.

### 3.6. ZJJ Treatment Improves the Astrocytic IR/IRS-1 Signaling in Diabetic Rats

**Figure 6:**
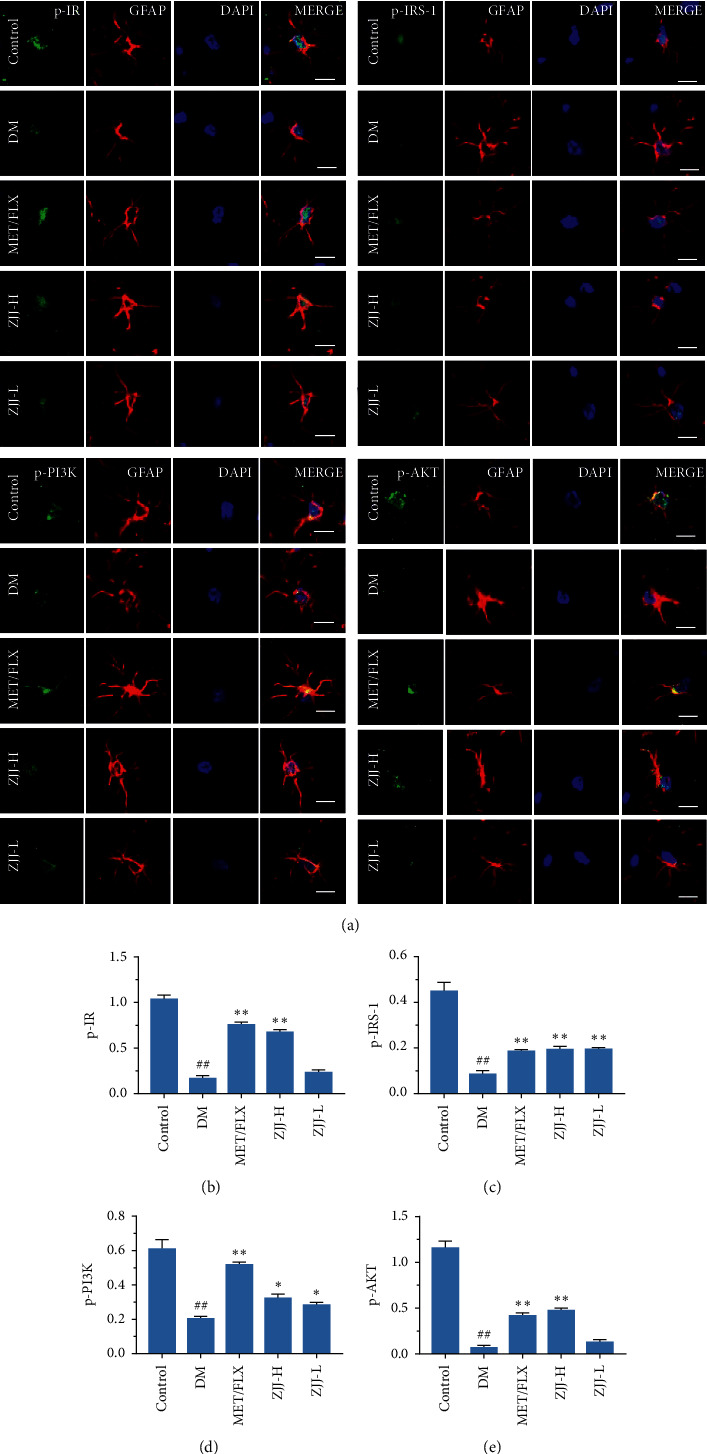
ZJJ activated the astrocytic insulin signaling pathway in diabetic rats. (a) Double immunofluorescence analysis of p-IR, p-IRS-1, p-PI3K, and p-AKT and a astrocytic marker (GFAP) in astrocyte. (b–e) The high dose of ZJJ treatment increased the immunopositivity of p-IR (b), p-IRS-1 (c), p-PI3K (d), and p-AKT (e), while the low-dose ZJJ treatment increased the expression of p-IRS-1 (c) and p-PI3K (d) in the hippocampus of diabetic rats. *n* = 5 in each group. Bar = 20 *μ*m. ^##^*P* < 0.01 vs. the control group. ^*∗∗*^*P* < 0.01 and ^*∗*^*P* < 0.05 vs. the DM group. Data are presented as mean ± SEM.

Given that insulin may act on astrocytes affecting both energy homeostasis and neurobehaviors, we further investigated the effect of ZJJ on astrocytic IR/IRS-1 signaling by using the double-labeling immunofluorescence method. As shown in Figure 6, compared with the control rats, p-IR, p-IRS-1, p-PI3K, and p-AKT expressions were significantly reduced in the astrocyte of diabetic rats (*P* < 0.01). After the high-dose (20.530 g/kg) ZJJ treatment, the expressions of p-IR, p-IRS-1, p-PI3K, and p-AKT were significantly increased (*P* < 0.05 or *P* < 0.01). And the low dose (10.265 g/kg) of this decoction obviously increased the p-IRS-1 and p-PI3K levels (*P* < 0.05 or *P* < 0.01).

### 3.7. ZJJ Treatment Modulates the Astrocytic Glycogen Metabolism in Diabetic Rats

**Figure 7:**
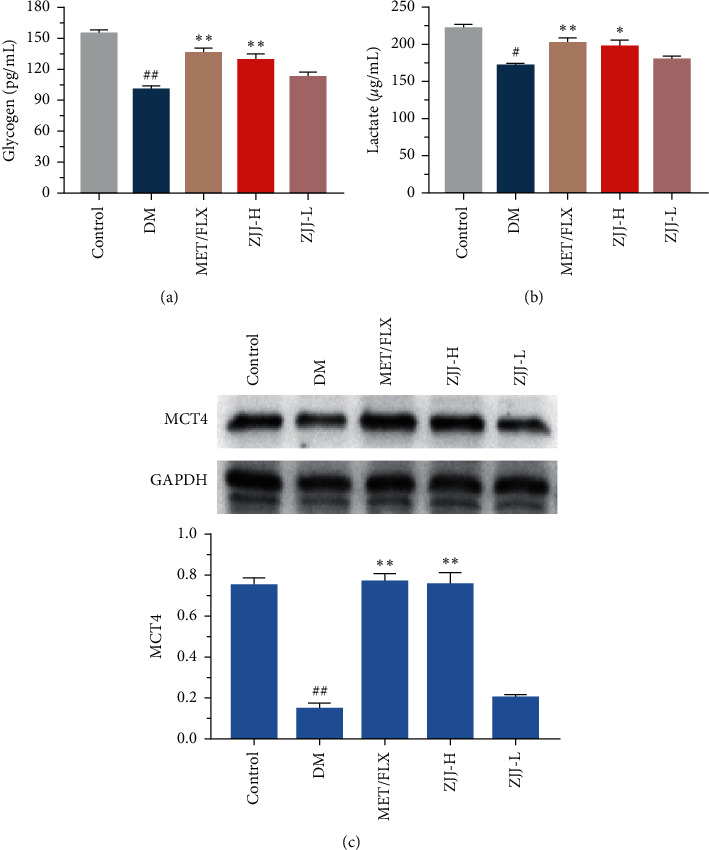
ZJJ improved the astrocytic glycogen metabolism in diabetic rats. (a, b) High dose of ZJJ increased the content of glycogen (a) and lactate (b) in diabetic rats. (c) Bands of MCT4 and GAPDH were shown, and the expression of MCT4 was increased after the high-dose ZJJ treatment in diabetic rats. *n* = 6 in each group. ^##^*P* < 0.01 and ^#^*P* < 0.05 vs. the control group. ^*∗∗*^*P* < 0.01 and ^*∗*^*P* < 0.05 vs. the DM group. Data are presented as the mean ± SEM.

The astrocyte glycogen and lactate play a key role in supporting neuronal function by providing energy [19]. As shown in Figure 7(a) and 7(b), the content of glycogen and lactate was reduced in the hippocampus of diabetic rats (*P* < 0.05 or *P* < 0.01). In addition, Western blot results indicate an obvious decrease in the expression of MCT4 in diabetic rats, which was responsible for exporting lactate out of the astrocyte (Figure 7(c), *P* < 0.01). ZJJ treatment increases the levels of glycogen and lactate, as well as the expression of MCT4 (*P* < 0.05 or *P* < 0.01).

## 4. Discussion

In this study, after 4 weeks of HFD and a single injection of STZ, SD rats exhibit periphery insulin resistance significantly. And the depression-like behavior along with cognitive dysfunction is found in diabetic rats after 12 weeks of diabetes duration. ZJJ shows the hypoglycemic and antidepression effect on diabetic rats with depression-like behavior, which is consistent with our previous studies [13]. Western blot and immunofluorescence methods demonstrate that ZJJ activates both neuronal and astrocytic IR/IRS-1 signaling pathways in the hippocampus of diabetic rats. Moreover, the decreased expression of GLUT4 and the content of ATP in diabetic rats are attenuated by ZJJ, indicating the potential capability of this decoction on neuronal energy metabolism. ZJJ upregulates the expression of MCT4, glycogen, and lactate, suggesting that astrocytic glycogen metabolism may participate in the function of ZJJ on diabetes-related depression. These findings provide evidence that ZJJ not only impacts on the energy metabolism by activating neuronal IR/IRS-1 signaling but also regulates the glycogen metabolism by stimulating astrocytic IR/IRS-1 signaling.

Notably, the method of chronic unpredictable mild stress (CUMS) is not used in diabetic rats in this study, which is different from the early studies. In fact, a clinical study reported that the duration of diabetes is associated with the depression in the later life of people with diabetes [20]. And, similarly, STZ-injected rats exhibit depression-like behavior after a period of diabetes [21]. Kamal et al. [22] reported that the damaged hippocampal synaptic plasticity, a key pathophysiological mechanism of depression, was observed after six- or eight-week duration of diabetes in STZ-induced diabetic rats. After 12 weeks of diabetes, the deficit of synaptic plasticity reaches the maximum and remains stable thereafter. Thus, the effect of ZJJ on depression-like behavior of diabetic rats was investigated without the interfering of CUMS in this study. Diabetic rats receive ZJJ administration for 4 weeks starting from the 8th week of diabetes. And the depression-like behavior and cognitive deficit in STZ-induced diabetic rats have been improved by ZJJ.

Recent years, some findings suggested that aberrant hippocampal IR/IRS-1 signaling was associated with depressive disorders [23, 24]. And mice with a brain-specific knockout of the insulin receptor (NIRKO mice) exhibit the age-related anxiety and depression-like behaviors [25]. In this study, phosphorylation of IR and IRS-1 was decreased in the hippocampus of diabetic rats. It is worth noting that there are numerous residues on IRS-1, including tyrosine, serine, and threonine. [26]. IR was activated by insulin, which then mediates different phosphorylation sites of IRS-1, while posing either positive or negative effects on insulin sensitivity. For instance, as a negative mediator of insulin signaling, ser(P)^307^-IRS-1 blocks the interaction between the IRS-1 and IR, which may lead to insulin resistance [27]. On the contrary, the increase in tyrosine phosphorylation of IRS-1, which is initiated by insulin, exhibited positive effects on insulin sensitivity in C57BL/6J mice [28]. In our previous study, the level of ser(P)^307^-IRS-1 was increased in the hippocampus of diabetic rats with depression [15]. And in this study, the level of tyr(P)-IRS-1 was decreased in the same brain area. Thus, the above findings suggest that aberrant IR/IRS-1 signaling exists in STZ-induced diabetic rats with depression-like behavior. In addition, the downstream molecules of IRS-1^tyr^, including PI3K and AKT, were likewise reduced in diabetic rats. Therefore, different sites of IRS-1 may involve in the brain insulin resistance in diabetic rats. And future research will be required to clarify the relationship between ser(P)-IRS-1 and tyr(P)-IRS-1 and their impact on the regulation of brain insulin resistance.

It is clear that the neuron is an insulin-sensitive cell. Insulin action on the neuronal cell has been shown to regulate systemic energy homeostasis [29]. Moreover, neuronal IR/IRS-1 signaling has been found to involve in some neurobehaviors including mood and cognitive function [30, 31]. In the present study, Western blot analysis in the present study supports that ZJJ activated the hippocampal insulin signaling pathway by increasing the phosphorylation of IR, IRS-1, PI3K, and AKT. In the meantime, immunofluorescence analysis shows that the expressions of p-IR, p-IRS-1, p-PI3K, and p-AKT were increased in hippocampal neurons, which was consistent with the result of Western blot method. In fact, the regulation on the neurobehavioral by IR/IRS-1 signaling could partly attribute to its regulation on brain glucose utilization [32], which plays an important role in the major depressive disorder. Grillo et al. [33] reported that the translocation stimulated by insulin of glucose transporter 4 (GLUT4) to the membrane rapidly increased glucose utilization of neurons. Furthermore, the process of GLUT4 translocated to the membrane is related with neurocognitive improvement [34]. On the contrary, impaired IR/IRS-1 signaling leads to the reduced levels of GLUT4 [35]. In the present study, ZJJ increased the GLUT4 expression in the hippocampus, implying the positive effect of this decoction on glucose uptake. In addition, the reduced ATP content in the hippocampus of diabetic rats was reversed by ZJJ administration. Therefore, ZJJ regulates energy metabolism in the hippocampus of diabetic rats with depression, which may be related to its regulation on neuronal IR/IRS-1 signaling.

Brain IR/IRS-1 signaling is important for energy homeostasis, cellular metabolism, and mood disorders, while most of these effects are thought to occur in neurons. As a matter of fact, brain cells include both neuron cells and glial cells. Notably, a previous study indicated that IR/IRS-1 signaling in the astrocyte is involved in regulating neural behaviors, including mood and cognitive function [9]. Besides, astrocytes might be a crucial target for IR/IRS-1 signaling. Interestingly, in this study, ZJJ has been found to regulate astrocytic IR/IRS-1 signaling by increasing the expression of major proteins. It is generally accepted that astrocytes are the main cells to store glycogen [36], which is involved in maintaining neuronal activity in the brain [19]. And IR/IRS-1 signaling could phosphorylate the downstream molecule AKT, which regulates the primary enzyme in glycogen synthesis [37, 38]. In this study, ZJJ increased the content of glycogen in astrocytes. This might be related to the function of ZJJ on the astrocytic IR/IRS-1 signaling pathway. Moreover, administration of ZJJ increases the levels of lactate, accompanied with increasing the expression of GLUT4. Indeed, when the energy demand exceeds the supply in the nervous system, glycogen is used to generate lactate, some of which is released from astrocytes by MCT4 and transported to the neighboring neurons [39]. Thus, the above findings suggest that ZJJ may improve the astrocytic glycogen metabolism to providing energy support for neurons in the hippocampus of diabetic rats with depression.

Consequently, our findings suggest that both neuronal and astrocytic IR/IRS-1 signaling pathways make important contribution to the treatment of ZJJ on diabetes-related depression. And we will further investigate the effect of ZJJ on the astrocyte-neuron metabolic coupling.

## Data Availability

The data sets used and/or analyzed during the current study are available from the corresponding author on reasonable request.

## Abbreviations

ZJJ: Zuogui Jiangtang Jieyu decoction
FST: Forced-swimming test
MWMT: Morris water maze test
IR: Insulin receptor
IRS-1: Insulin receptor substrate-1
PI3K: Phosphatidylonositol-3-kinase
AKT: Protein kinase B
STZ: Streptozotocin
GFAP: Glial fibrillary acidic protein
HPA: Hypothalamic-pituitary-adrenal
GLUT4: Glucose transporter 4
MCT4: Monocarboxylate transporter 4.
